# Mechanism of Protection Induced by *Group A Streptococcus* Vaccine Candidate J8-DT: Contribution of B and T-Cells Towards Protection

**DOI:** 10.1371/journal.pone.0005147

**Published:** 2009-04-02

**Authors:** Manisha Pandey, Michael R. Batzloff, Michael F. Good

**Affiliations:** The Australian Centre for Vaccine Development, The Queensland Institute of Medical Research, PO Royal Brisbane Hospital, Brisbane, Australia; Columbia University, United States of America

## Abstract

Vaccination with J8-DT, a leading GAS vaccine candidate, results in protective immunity in mice. Analysis of immunologic correlates of protection indicated a role of J8-specific antibodies that were induced post-immunization. In the present study, several independent experimental approaches were employed to investigate the protective immunological mechanisms involved in J8-DT-mediated immunity. These approaches included the passive transfer of mouse or rabbit immune serum/antibodies in addition to selective depletion of T-cell subsets prior to bacterial challenge. Passive transfer of J8-DT antiserum/antibodies from mice and rabbits conferred significant resistance against challenge to mice. To exclude the possibility of involvement of other host immune factors, the studies were repeated in SCID mice, which highlighted the need for an ongoing immune response for long-lived protection. Depletion of CD4^+^ and CD8^+^ T-cell subsets confirmed that an active *de novo* immune response, involving CD4^+^ T-helper cells, is required for continued synthesis of antibodies resulting in protection against GAS infection. Taken together these results indicate an involvement of CD4^+^ T-cells in J8-DT-mediated protection possibly via an ability to maintain antibody levels. These results have considerable relevance to the development of a broad spectrum passive immunotherapy for GAS disease.

## Introduction

Group A streptococcus (GAS) is a Gram-positive bacterial pathogen responsible for a wide variety of diseases ranging from self-limiting generally benign conditions such as streptopcoccal pharyngitis and pyoderma to invasive diseases including necrotising fasciitis. However, of most concern are the post infectious sequelae of rheumatic fever/rheumatic heart disease and chronic renal disease. To date, GAS vaccine development has primarily focussed on the M-protein. The M-protein is a cell surface protein that is highly variable at the amino terminus (serotypic determinants) but is highly conserved at the carboxyl terminus.

Development of a vaccine for group A streptococcus (GAS), has been hampered due to a number of serotypic variants of the M-protein and the possibility of cross-reactivity of potential vaccine candidates with host tissues. However, we have previously identified a minimal B-cell epitope from the conserved region of M-protein of GAS and demonstrated its protective potential [1], [2]. The immune response to this minimal B-cell epitope, referred to as J8, was found to be genetically restricted and J8 was nonimmunogenic in an outbred population. To overcome this non-responsiveness, the peptide J8 was conjugated to the carrier protein, diphtheria toxoid (DT) [3]. Carrier proteins such as DT stimulate T-helper cells which co-operate with B-cells to enable them to respond to the hapten by providing accessory signals.

The conjugated vaccine candidate, J8-DT, formulated with the human compatible adjuvant, alum, was shown to be protective in inbred and outbred mouse strains [3]. Evidence that antibodies had a role in vaccine mediated immunity came from the observed positive correlation between high J8-specific IgG titres and mouse survival [3]. Therefore, the J8-specific antibody response was considered to be important in protection against GAS infection.

A passive transfer system has been used in this study to confirm the role of antibodies in protection and also to investigate the potential therapeutic utility of antibodies for treating GAS infections. Passive immunization with antibodies or antiserum has been shown to be protective in a number of viral, bacterial and parasitic models [4]–[8]. Therapeutic antibodies may play an important role in treating infections caused by drug resistant pathogens as well as pathogens for which no antimicrobial drug is available. Outcomes of clinical studies to date using IVIG treatment for invasive GAS disease have been variable [9], [10]. The sources of IVIG for these studies vary and levels of anti-streptococcal antibodies poorly defined. Furthermore, the immunocompetence of the recipients and what effect if any, this may have on the clinical outcome of the therapy has not been ascertained. To investigate the potential of J8-specific antibodies and the role of the recipient's immune system in any therapeutic effect, a passive transfer system was studied in a mouse model in which antibody dosing and the role of host T and B lymphocytes were determined.

## Materials and Methods

### I. Mice

Four to six week old female BALB/c, B10.BR or SCID mice were purchased from The Animal Resource Centre, Perth, Western Australia. All animal protocols used were approved by the Institute's ethics committee (Queensland Institute of Medical Research Animal Ethics Committee) in accordance with National Health and Medical Research Council (NHMRC) of Australia guidelines.

### II. Peptide synthesis and conjugation to a peptide carrier

The peptide J8 (sequence QAEDKVKQSREAKKQVEKALKQLEDKVQC) was synthesised as described elsewhere [11] and purified using high-performance liquid chromatography. Peptide was conjugated via a C-terminal cysteine residue to DT (CSL, Australia), using 6′-maleimido-caproyl n-hydroxy succinimide (MCS), as described by Coligan et al. [12].

### III. Immunization of mice

Cohorts of 20–30 BALB/c mice were subcutaneously immunized at the tail base on day 0 with 30 ug of J8-DT or DT adsorbed on alum. The antigens diluted in PBS were adsorbed onto alum at room-temperature for one hour with slow mixing before being injected into mice. To control for the effect of the adjuvant, parallel cohorts of mice were given PBS in alum. All the groups also received three subsequent boosts on day 21, 28 and 35. In some studies B10.BR mice (inbred H-2^k^ background) were used for J8 immunization. Peptides (peptide alone or peptide conjugated to DT) were administered subcutaneously in a volume of 50 ul at the tail base to B.10.BR mice. Each mouse received a total of 30 ug of immunogen (free peptide or conjugated peptide) emulsified 1∶1 in CFA (Sigma, USA). Control mice received PBS emulsified in CFA (PBS/CFA). All the groups also received three subsequent boosts on day 21, 28 and 35 in PBS. In some studies, post immunization, mice were depleted of CD4^+^ or CD8^+^ T-cells prior to challenge with GAS. Serum samples were collected on day 20, 27, 34 and 42 and IgG concentrations and/or titers measured by ELISA [2].

### IV. Preparation of J8 or J8-DT immune serum

High titer (titer >10^6^) immune sera were collected from immunized and control mice periodically. A week after the last boost (day 42), the mice were bled by cardiac puncture. The blood was allowed to clot at room temperature for 30 minutes followed by overnight storage at 4°C to enable the clot to retract. The clot was removed and supernatant was spun at 3000 rpm for 10 minutes. After spinning, the serum was collected and stored at −20°C until used. To avoid antibody aggregation, the serum samples were sonicated under cold conditions before being transferred into the recipient mice [13].

### V. Passive transfer of immune serum and GAS challenge

The pooled serum (from each group) was transferred intraperitoneally into the BALB/c, SCID or B10.BR mice in three doses of 0.5 ml each on day −1, 0 and +1 relative to the day of challenge. Some cohorts of SCID mice received additional doses that were administered post-challenge on day 3, 5 and 8. Two hours after the second administration of antiserum/antibodies, on day 0, serum samples were collected to confirm the success of serum transfer by measuring antigen specific IgG levels in the recipient mice.

On day 0 the recipient mice were challenged intraperitoneally with a predetermined dose of M1 GAS as described previously [3]. Following challenge, the mice were closely observed and their survival monitored on a regular basis for 10–15 days.

### VI. Production, purification and passive transfer of rabbit IgG

Antibodies to J8-DT or to DT were raised in rabbits at IMVS, Adelaide, South Australia. Two rabbits (New Zealand white, males 6–8 weeks) were vaccinated subcutaneously multiple times using 0.5 mg of J8-DT or DT antigen preparation in alum. Following primary immunization, four subsequent boosts were given at monthly intervals and serum samples were collected to measure antibodies to J8. At the end of the boosting period a terminal bleed was conducted and serum samples were used to purify IgG antibodies as described below.

Rabbit IgG were purified using protein-G sepharose column (GE Healthcare, USA). Briefly, rabbit antisera were diluted 1∶2 and passed through protein G columns enabling antibodies to bind to the column. The antibodies were then eluted using glycine-HCl buffer (pH 2.7), neutralized using Tris-HCl (pH 9.0), dialysed and concentrated. The concentration of total IgG was quantified in the purified preparation and an amount equivalent to what was present in 500 ul of mouse antiserum were administered intraperitoneally into mice on three consecutive days (day −1, 0 and +1) in a volume of 500 ul each. The mice were challenged with M1 GAS on day 0.

### VII. ELISA

ELISAs were performed for antibody determination as essentially described [2]. NUNC immunoplates (Flow laboratories) were coated with 100 ul of J8 or DT at 5 ug/ml in carbonate-bicarbonate buffer (pH 9.6), overnight at 4°C, as previously standardised in our laboratory. Serum samples were assayed by plating 2-fold dilutions of a 1∶100 dilution of serum. The end point titers were determined as the highest dilution of serum for which the OD was 3SD above the mean OD of control wells containing serum from naïve mice. For quantitative ELISA, standard curves were plotted using purified IgG preparations and the IgG concentrations in unknown samples were determined [14]. The concentrations of various IgG isotypes were also determined.

### VIII. In vivo CD4^+^ and CD8^+^ T- cell depletion

One week after the last boost with J8-DT or DT, selected cohorts of mice received 0.3 mg of rat anti-CD4 (GK1.5) or 1 mg of anti-CD8 (ά-CD8-beta clone 53.5.8) mAb intraperitoneally before (D−2) and after (D+1, +4, +7) a GAS challenge. The dose and time-course for both depletions were previously optimised using FACS to determine the degree of cell depletion (data not shown). The schedule resulted in greater than 99% depletion of CD4^+^ T-cells and 95 to 97% deletion of CD8^+^ T-cells as assessed by FACS analysis. Control groups included J8-DT immunized mice, which were untreated or treated with normal rat IgG (nRIg), and PBS immunized mice which were treated with anti-CD4/anti-CD8 mAb or nRIg.

### Statistical Analysis

The geometric mean and SEM were calculated using standard formulas. Mann-Whitney test was used to compare the proportions of surviving mice challenged with GAS. P<0.05 was considered to be statistically significant.

## Results

### 1. Antibody/isotypes response to immunogen

**Figure 1:**
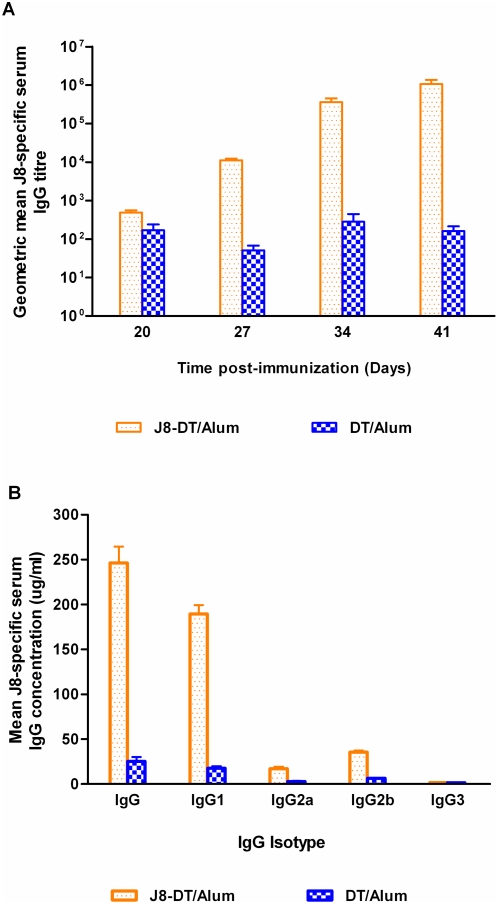
J8-specific serum IgG antibody response in BALB/c mice immunized parenterally with J8-DT or DT. Cohorts of BALB/c mice (n = 10/group) received a primary immunization on day 0 followed by three boosts on day 21, 28 and 35. Serum samples were collected before each vaccination and one week after last boost. The geometric mean titres (GMT) of J8-specific serum IgG (a) and mean concentrations of various J8-specific IgG isotypes in the final bleed serum of BALB/c mice (b), determined by ELISA are shown.

Immunization of BALB/c mice with J8-DT/alum induced production of IgG antibodies that recognised J8 peptide. As expected, boosting of mice with the same immunogen resulted in an increase in J8-specific IgG titres (Figure 1a). After three boosts, J8-specific IgG titers were approximately 10^6^ and sera were used for passive transfer. Immunization with DT produced high level of anti-DT IgG which showed no specificity for the J8 peptide in ELISA. To further characterize the antibody responses, antibody isotypes were quantified. Immunization of BALB/c mice with J8-DT/alum induced significantly higher level of IgG1 than of IgG2a or IgG2b. In general, the levels of IgG3 were undetectable (Figure 1b).

### 2. Passive transfer of immunity in naive mice by J8-DT antisera

#### 2.1 Passive transfer of murine J8-DT antisera into BALB/c mice

**Figure 2:**
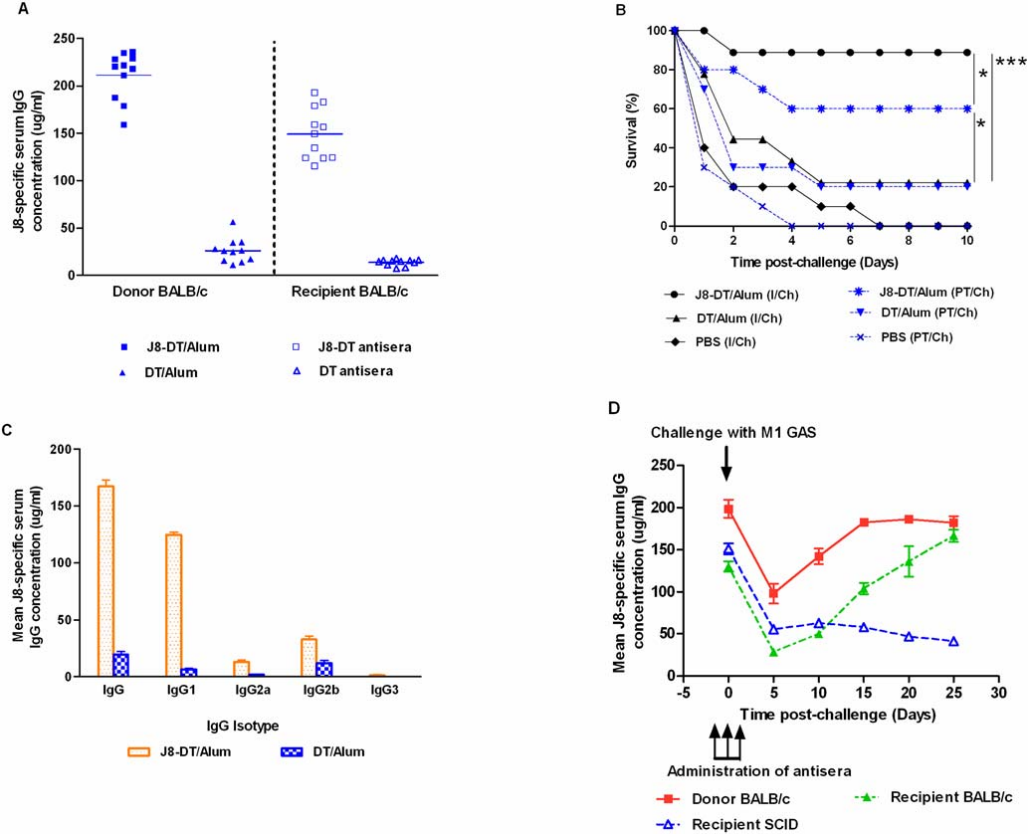
J8-specific serum IgG response and survival in mice following active and passive immunization. (a) J8-specific IgG concentrations in serum of individual donor and recipient mice are shown, with mean concentration represented as horizontal bars. (b) Survival following M1 GAS challenge in BALB/c mice after active (I/Ch) and/or passive (PT/Ch) immunization. Mice were challenged intraperitoneally with M1 GAS and their survival monitored for 10–12 days. Mann-Whitney test was used to compare the proportions of surviving mice. Significance is represented as * p<0.05 and *** p<0.001. (c) Mean concentration of various J8-specific IgG isotypes in the serum of recipient BALB/c mice that received J8-DT or DT antiserum. (d) J8-specific mean serum IgG concentrations in donor (BALB/c) and J8-DT antiserum recipient (BALB/c and SCID) mice at different time-points post active and passive immunization followed by a lethal challenge.

In order to determine the protective efficacy of antibody, J8-DT, DT or PBS antisera were passively transferred into naïve mice, which were subsequently challenged with M1 GAS. First we confirmed that two injections of antiserum would result in an immediate titre of antibody in the recipients. As depicted in Figure 2a, all recipient mice had high levels of anti-J8 IgG antibodies when tested on day 0 after the second serum administration. Following a lethal challenge, none of the immune mouse serum recipients nor the immunized mice showed any sign of illness during the first 24 hrs of infection. On the contrary, the control mice that received DT or PBS antisera showed signs of morbidity (ruffled fur, lack of response to external stimulus, hunched posture). Passive transfer of J8-DT antisera into BALB/c mice significantly protected them from a lethal GAS challenge compared to mice that received DT antiserum (p<0.05) (Figure 2b). Mice receiving DT antisera did not have significantly higher rates of survival (p>0.05) and died within 5 days of challenge as did the controls that received PBS antisera. In contrast mice receiving J8-DT antisera (with an end point J8-specific IgG concentration of at least 100 ug/ml or more) were protected against a lethal challenge of GAS. Antibody isotyping showed a preponderance of IgG1 in transferred mouse serum (Figure 2c). The lower level of protection (60%) in J8-DT antiserum recipients compared to vaccinated-challenged controls (80%) suggested a possible involvement of some other factors beside transferred antibodies in vaccine mediated protection. We also noted that the level of J8-specific IgG fell dramatically soon after challenge both in donor and antiserum recipient mice (Figure 2d). However, donor immunized mice having their immune system primed through immunization, recover more efficiently compared to antiserum recipient mice which is suggestive of an ongoing active immune response in the host.

#### 2.2 Passive transfer of J8-DT antisera to SCID mice

**Figure 3:**
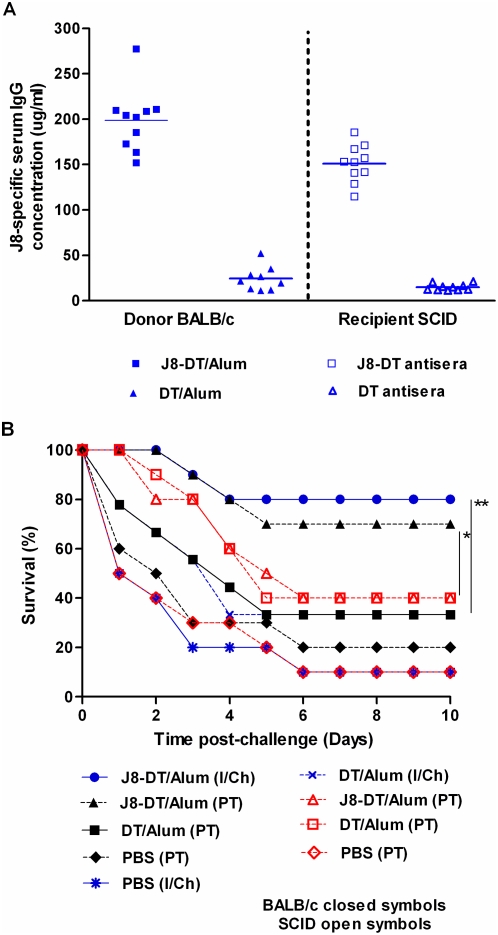
Antibody levels and percent survival of BALB/c and SCID mice following active and/or passive immunization with J8-DT, DT or PBS. (a) J8-specific serum IgG concentrations in donor BALB/c or recipient SCID mice. Serum samples were collected post-immunization (for donors) or post second administration of J8-DT or DT antiserum (for recipients). J8-specific IgG concentrations in serum of individual mice, are shown, with mean concentration represented as horizontal bars. (b) Protection induced in BALB/c and SCID mice by J8-DT following active (I/Ch) and/or passive immunization (PT/Ch). The mice were challenged with M1 GAS and their survival monitored. Mann-Whitney test was performed to compare the proportions of surviving mice. Significance is represented as * p<0.05 and ** p<0.01.

To determine whether a *de novo* immune response of the host was required for protection following passive transfer, these studies were repeated in immunocompromised SCID mice (deficient in both B and T-cells). J8 and DT-specific antibodies were found in the serum of SCID mice post transfer (Figure 3a). Our results demonstrated that BALB/c mice receiving J8-DT antiserum survived significantly longer (p<0.05) than corresponding SCID mice or BALB/C mice receiving DT antiserum (Figure 3b). However, both mouse strains had similar level of passively transferred IgG (Figures 2a, 3a). We noted that the majority of the antibodies were consumed both in BALB/c and SCID recipient mice soon after bacterial challenge (Figure 2d) but BALB/c mice were able to continue synthesizing adequate levels of IgG (as demonstrated by ELISA, Figure 2d). These data suggest that an ongoing immune response may be required to protect mice following passive transfer. The low level of protection offered by DT antiserum was not significantly higher compared to PBS antiserum recipient groups (p>0.05).

### 3. Antibodies to J8 are protective without the involvement of DT

**Figure 4:**
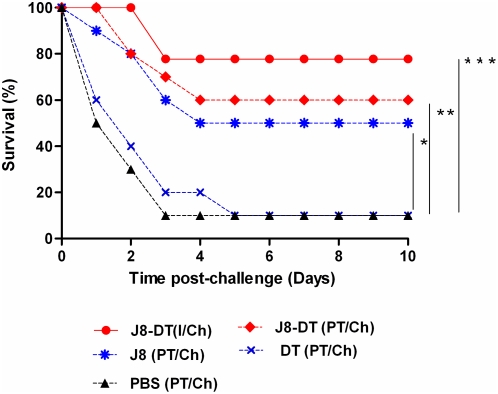
Protection induced in B10.BR mice following passive administration of J8/J8-DT/DT or PBS antisera from B10.BR mice. Cohorts of B10.BR mice received antisera from mice immunized with J8/J8-DT/DT or PBS in CFA. The antisera was passively administered to B10.BR mice on three consecutive days (day −1, 0 and +1). The mice were challenged with M1 GAS on day 0 and their survival monitored. I/Ch represent the cohort of mice that were immunized with J8-DT. Significance is represented as * p<0.05, ** p<0.01 and *** p<0.001.

All the studies discussed so far have used J8-DT antibodies and demonstrated their role in protection against GAS. To confirm that J8 antisera are protective, experiments involving passive transfer of J8 antiserum were conducted (Figure 4). We have shown previously that B10.BR mice can respond to J8 alone [2]. Serum collected from J8/CFA or J8-DT/CFA immunized B10.BR mice were passively transferred to naive recipient B10.BR mice. This resulted in equivalent levels of protection after a GAS challenge.

### 4. IgG is responsible for passive transfer of protection

**Figure 5:**
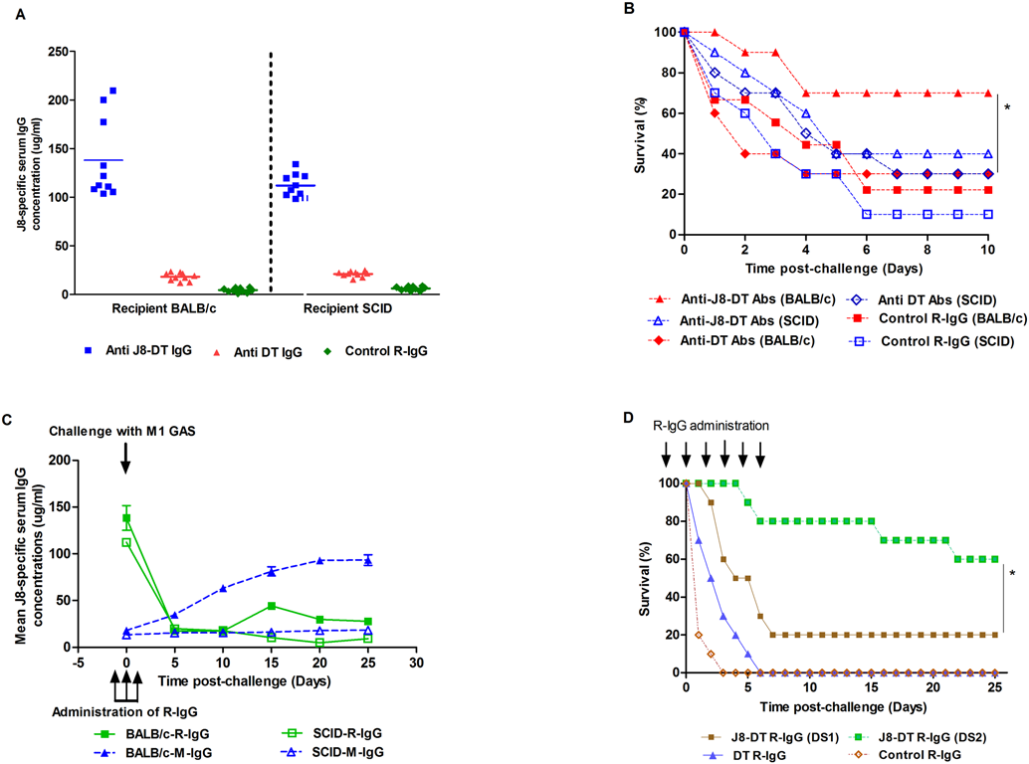
J8-specific IgG concentrations and protection in BALB/c and SCID mice following passive transfer of purified rabbit IgG. Rabbits were multiply vaccinated with J8-DT and DT preparations in alum. Purified IgG (0.5 mg) was administered intraperitoneally into BALB/c and SCID mice on each of three days (day −1, 0 and +1). Controls received similar amount of normal rabbit IgG (control R-IgG). The mice were challenged on day 0 with M1 GAS and their survival monitored. (a) Demonstrates the concentration of J8-specific serum IgG in recipient BALB/c and SCID mice post intraperitoneal transfer. (b) Survival of BALB/c and SCID mice following a M1 GAS challenge (significance is represented as * where p<0.05) (c) Shows the concentration of J8-specific mouse (M) and rabbit (R) IgG in the serum of recipient mice at different time-points post antibody transfer followed by M1 GAS challenge and (d) shows survival in SCID mice following additional doses (on day 3, 5 and 8) of J8-DT R-IgG or control R-IgG post GAS challenge. The abbreviation DS1 represents dose schedule 1 (day −1, 0 and +1) whereas DS2 represents dose schedule 2 (Day −1, 0, +1, +3, +5 and 8). The mice were challenged on day 0 with M1 GAS and their survival monitored. Significance is represented as * where p<0.05.

To demonstrate that IgG is transferring protection, affinity purified J8-DT or DT rabbit IgG were transferred passively into BALB/c and SCID mice. Each naïve mouse received three doses of 500 ug of rabbit IgG (from J8-DT or DT immunized rabbits) (Figure 5a). Post-challenge, the mice receiving rabbit J8-DT IgG (J8-DT R-IgG) were significantly protected (p<0.05) compared to control mice receiving DT-specific IgG or normal rabbit IgG. Groups of mice that received DT-specific IgG did not have significantly higher rates of survival than did groups that received rabbit IgG (p>0.05) (Figure 5b). We found that 500 ug J8-DT R-IgG provided better protection than 250 ug or 125 ug of J8-DT R-IgG (data not shown). IgG recipient BALB/c mice were better protected than the IgG recipient SCID mice (Figure 5b). We again noted that following challenge, as the passively transferred rabbit antibodies were being depleted, the active host immune response in BALB/c mice commenced resulting in the production of J8-specific antibodies (Figure 5c). As expected this was not observed in SCID mice. However, additional doses of R-J8-DT IgG were able to significantly protect SCID mice compared to DT or control IgG. The majority of the SCID mice in the cohort that received routine scheduled doses of IgG (day −1, 0 and +1) succumbed to infection by day 10 (20% survival) in contrast to 80% which survived after receiving additional doses of IgG on the day 3, 5 and 8 (Figure 5d).

### 5. Requirement of T-cells for enhanced protection

#### 5.1 Role of CD4^+^ T-cells in protection induced by J8-DT

To determine whether T-cells were involved in protection, CD4^+^ T-cells were depleted from BALB/c mice post-immunization by treatment with GK1.5 mAb before and after challenge. This protocol resulted in 99% depletion (data not shown).

**Figure 6:**
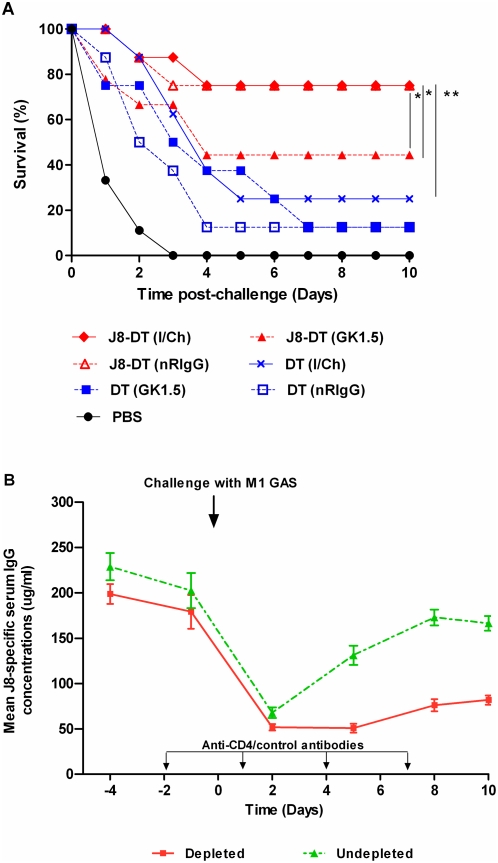
Antibody levels and percent survival of BALB/c mice immunized and depleted/undepleted of CD4^+^ T-cells. BALB/c mice were immunized with J8-DT/DT or PBS parenterally. For *in vivo* depletion of CD4^+^ T-cells mice were administered with 0.3 mg of anti-CD4 (GK1.5) antibodies intraperitoneally over a set time-course before and after challenge as shown. Protection (a) and antibody concentrations before and after challenge (b) in J8-DT immunized and CD4^+^ T-cell depleted/undepleted BALB/c mice are shown. The abbreviation I/Ch stands for immunized/undepleted mice (positive controls). Significance is represented as * p<0.05 and ** p<0.01.

Following GAS challenge, PBS immunized mice untreated or that had been treated with GK1.5 or control antibodies (nRIgG) experienced a fulminant infection and succumbed within three to four days post infection (Figure 6a). In contrast, J8-DT immunized mice that were depleted of CD4^+^ T-cell had reduced protection (44% survivors) compared to J8-DT immunized untreated mice (75% survivors) or mice treated with nRIgG (75% survivors). Mice in the DT immunized group, whether treated with GK1.5 or nRIgG, had lower survival (12.5%) which was not significantly higher (p>0.05) compared to PBS control. These data suggest that an active immune response involving CD4^+^ T-cells is required for protection.

We further observed that by day 2 post-challenge antibody levels had dropped dramatically in CD4^+^ T-cell depleted, J8-DT immunized mice and did not recover. In contrast, J8-DT immunized mice either treated with nRIgG or untreated were capable of generating antibodies following challenge (Figure 6b).

#### 5.2 Role of CD8+ T-cells in protection induced by J8-DT

**Figure 7:**
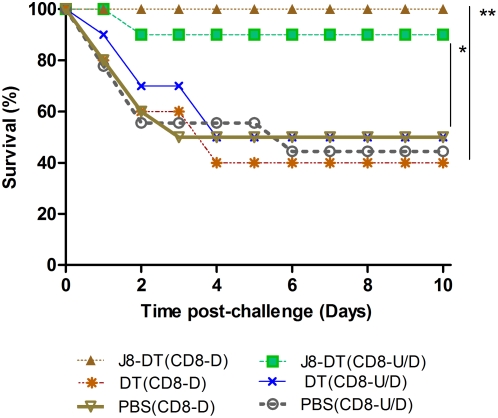
Survival of BALB/c mice immunized and depleted/undepleted of CD8^+^ T-cells. BALB/c mice were immunized with J8-DT/DT or PBS parenterally. For *in vivo* depletion of CD8^+^ T-cells mice were administered with 1 mg of anti-CD8 (α-CD8-beta clone 53.5.8) mAb intraperitoneally over a set time-course before and after GAS challenge. Survival in J8-DT immunized and CD8^+^ T-cell depleted/undepleted BALB/c mice are shown. The abbreviation D stands for immunized/depleted mice whereas U/D represents immunized/undepleted. Significance is represented as * p<0.05 and ** p<0.01.

Next, J8-DT immunized BALB/c mice were depleted of CD8^+^ T-cells and were subsequently challenged with M1 GAS. The protocol resulted in >95% depletion of CD8^+^ T-cells (data not shown). Depletion of CD8^+^ T-cell subset did not diminish protection (Figure 7).

## Discussion

In this study we have investigated the role of humoral and cell mediated immune responses in protection induced by the GAS vaccine candidate, J8-DT. We have previously shown that immunization with J8-DT adsorbed onto alum induced high levels of J8-specific antibodies, which were capable of protecting outbred mice from a lethal challenge with GAS [3]. Here again we have been able to reproduce similar results using inbred BALB/c (H-2^d^ background) mice. This observation suggested to us that the antibody response is an integral component of the protection induced by J8-DT. Since previous studies were performed in immunocompetent mice, we had not been able to exclude the possibility of involvement of T-cells in protection. In this study we have taken two different approaches to dissect the roles of antibodies and T-cells in protection: (i) passive transfer of antibodies into immunocompetent (BALB/c and B10.BR) and immunocompromised (SCID) mice, and, (ii) selective depletion of T-cell subsets (CD4^+^ and CD8^+^ T-cells) following immunization.

Passive immunity has been investigated for a number of organisms [4]–[8]. In the case of extracellular bacteria, passively transferred antibodies are believed to be involved in agglutination of bacteria or in alteration of the dissemination pattern of bacteria from the site of inoculation [15]. For intracellular bacteria such as Listeria and Mycobacteria, previous studies have indicated that specific antibodies have little, if any role in protection [15]. In contrast, some recent studies have shown that antibodies can be effective against intracellular infection as in the case of *E. chaffeensis* [16]–[18]. These studies suggest that the bacteria may not always reside in the intracellular space and thus could become accessible to serum antibodies.

We observed that passive transfer of J8-DT antisera into naïve recipient mice resulted in significantly increased survival compared to mice receiving DT antisera. However, the levels of protection in passively immunized mice were never as high as in actively immunized controls. This could be explained by the presence of long-lived plasma cells (LLPC) and continuous antibody synthesis in actively immunized mice. However, the diminished protection observed in SCID mice compared to BALB/c mice could be explained by the deficiency of B and T-cells in these mice and their inability to mount a specific response to the bacterial challenge following depletion of the passively transferred antibodies. Of interest was the observation that SCID mice demonstrated enhanced protection following transfer of additional doses of rabbit J8-DT IgG.

It has been reported that efficient protection after vaccination could only be acquired by elicitation of high level of long-lasting anti-GAS specific antibodies [19]. Polyclonal antisera raised against heat killed GAS was capable of transferring passive protection which was dependent on the amount of anti-GAS antibodies present in the immune serum and the time of administration post-infection. Our previous studies have also suggested that high levels of anti-J8 antibodies are required for protection [3].

The enhanced protection in actively immunized mice and reduced protection in passively immunized SCID mice suggested that T-cells may play a major role in vaccine mediated protection. It has been reported that CD4^+^ T-cells are important for development of long term immunity to bacterial infections [20]. Development of the appropriate CD4^+^ T-cell subset during an immune response is critical for eradication of an infectious organism. In vaccine mediated protection CD4^+^ T-cells are necessary to provide help to B-cells and CD8^+^ T-cells, as well as having effector function of their own in some situations. The CD4^+^ T-cells bind to the epitopes presented by B-cells which results in the development of clones of plasma cells secreting antibodies against the antigenic material. We have observed here that immunized mice, when depleted of CD4^+^ T-cells have reduced level of protection, suggesting that CD4^+^ (helper) T-cells are important for vaccine mediated protection. It has been shown in the case of rabies vaccine that passive antibody alone is poorly effective unless supplemented by transfer of CD4^+^ and CD8^+^ T-cells [21], [22]. Similar findings have been reported in the case of a malaria vaccine candidate where an absolute requirement of CD4^+^ T-cells was observed to enhance passive immunity [23]. In contrast, previous studies involving *S. pneumoniae* demonstrated CD4^+^ T-cell independent passive protection. [15]. These studies utilized polysaccharide antigens which are known to be thymus independent and have ability to stimulate B-cells directly. In this study the conjugation of J8 to DT generates a T-cell dependent antibody response that leads to the production of protective antibodies and possibly immunologic memory.

In summary, we have demonstrated that purified IgG from J8-DT immunized donor animals can protect naïve recipient mice including immunocompromised SCID mice. Taken together these data demonstrates the potential utility of J8-specific IgG in passive immunotherapy for the treatment of GAS diseases.
